# Renal damage-induced hepcidin accumulation contributes to anemia in angiotensinogen-deficient mice

**DOI:** 10.1042/CS20241789

**Published:** 2025-02-07

**Authors:** André F. Rodrigues, Laura Boreggio, Tetiana Lahuta, Fatimunnisa Qadri, Natalia Alenina, Carlos C. Barros, Mihail Todiras, Michael Bader

**Affiliations:** 1 Max-Delbrück-Center for Molecular Medicine in the Helmholtz Association (MDC), Berlin, Germany; 2 German Center for Cardiovascular Research (DZHK), Partner Site Berlin, Germany; 3 Charité Universitätsmedizin Berlin, Berlin, Germany; 4 Bogomoletz Institute of Physiology, Department of General and Molecular Pathophysiology, NAS of Ukraine, Kyiv, Ukraine; 5 Nutrition Faculty, Federal University of Pelotas - UFPel, Pelotas, RS, Brazil; 6 Technological Development Center, Federal University of Pelotas - UFPel, Pelotas, RS, Brazil; 7 Nicolae Testemițanu State University of Medicine and Pharmacy, Chisinau, Moldova; 8 Experimental and Clinical Research Center, a cooperation between the Max-Delbrück-Center for Molecular Medicine in the Helmholtz Association and the Charité - Universitätsmedizin Berlin, Berlin, Germany; 9 Institute for Biology, University of Lübeck, Lübeck, Germany

**Keywords:** anemia, chronic kidney, diseaserenin-angiotensin system

## Abstract

Angiotensin II (Ang II) is the most active peptide hormone produced by the renin–angiotensin system (RAS). Genetic deletion of genes that ultimately restrict Ang II formation has been shown to result in marked anemia in mice. In this study, adult mice with a genetic deletion of the RAS precursor protein angiotensinogen (Agt-KO) were used. Experimental analyses included capillary hematocrit, hemogram, plasma and tissue iron quantifications, expression analyses of genes encoding relevant proteins for body iron homeostasis in different organs, as well as plasma and urine hepcidin quantifications. As previously reported, Agt-KO were anemic with reduced red blood cell counts. Interestingly, we found that they presented microcytic anemia based on the reduced red blood cell volume. In agreement, plasma quantification of iron revealed lower levels of circulating iron in Agt-KO. The major body iron stores, namely in the liver and spleen, were also depleted in the RAS-deficient line. Hepatic hepcidin expression was reduced, as well as one of its major regulators, BMP6, as a result of the iron deficiency. However, plasma hepcidin levels were unexpectedly increased in Agt-KO. We confirm the typical morphological alterations and impaired renal function of Agt-KO and conclude that hepcidin accumulates in the circulation due to the reduced glomerular filtration in Agt-KO, and therefore identified the culprit of iron deficiency in Agt-KO. Collectively, the data demonstrated that the severe anemia developed in RAS-deficient mice is exacerbated by iron deficiency which is secondary to the renal damage-induced hepcidin accumulation in the circulation.

## Introduction

Angiotensin II (Ang II) is the primary active peptide hormone of the renin–angiotensin system (RAS) with well-characterized roles in blood pressure and fluid balance [1–3]. Ang II is also known as a modulator of erythropoiesis [3–5]. There is evidence showing that Ang II acts as a growth factor on bone marrow erythroid progenitors [6,7]. Other studies demonstrated that Ang II increases erythropoiesis by facilitating the expression/release of the major cytokine involved in erythropoiesis regulation, erythropoietin, from renal fibroblasts [8–10]. More recently, we demonstrated that brain Ang II modulates bone marrow sympathetic activity and indirectly regulates erythropoiesis [11].

Several studies in humans and preclinical models demonstrated that the angiotensin type 1 receptor (AT1R) mediates the effects on erythropoiesis triggered by Ang II. In rodents, the AT1aR (*Agtr1a*) is the predominantly expressed AT1R and its relevance for cardiovascular control is comparable to the only AT1R (*AGTR1*) encoded by the human genome [1,3]. Rodents have an additional gene encoding the AT1R (AT1bR, *Agtr1b*); however, this gene has a minimal influence on cardiovascular homeostasis in comparison with the AT1aR. For the control of erythropoiesis *in vivo*, both receptors seem to be equally important, because the genetic deletion of either AT1aR or AT1bR in mice does not cause anemia, only if both AT1Rs (AT1aR and AT1bR) are *knocked out* in parallel, the phenotype is observed [5]. In addition, mice lacking angiotensinogen, renin, or angiotensin-converting enzyme (ACE) are anemic [4,5,12].

In this study, we confirmed anemia in RAS deficiency using Agt-KO mice. Moreover, we uncover a phenotype not yet observed, namely iron deficiency, which contributes to the anemia observed in the absence of the Ang II/AT1R signaling. Agt-KO presented lower levels of iron in plasma and tissues. Consequently, hepatic hepcidin mRNA levels were found reduced to facilitate duodenal iron absorption. Contrary to the mRNA levels, plasma circulating levels of hepcidin were increased due to reduced glomerular filtration rate (GFR). Altogether the data show that the anemic phenotype often found in RAS-deficient mouse strains is partially based on the developmental renal damage typically observed in these lines. The data also confirm that, in case of reduced GFR, urinary hepcidin excretion may be significantly impaired leading to iron deficiency.

## Methods

### Animals

Agt-KO was generated by Tanimoto et al*.* [13] in a mixed background strain. We have backcrossed this line to the inbred FVB/N genetic background as described in Rodrigues et al*.* [14] All mice were kept in ventilated cages at 21°C ± 1°C [3–6] in each cage and had free access to standard chow and water. Most of the experiments were carried out with adult males aged between 12 and 15 weeks. Some of the experiments were performed with females of the same age. Heterozygous littermates (Agt-Het) were used as controls. Littermates from different genotypes were kept in the same cage to avoid cofounders. *In vivo* animal procedures were previously approved by the local authority (LAGeSo, Landesamt für Gesundheit und Soziales, #X9003/16 and #X9003/22) and were performed in agreement with what proposed by the ARRIVE guidelines. All experiments with animals were carried out at the Max Delbrück Center for Molecular Medicine, Berlin, Germany.

### Biological sample collection

Spontaneous urine was collected by holding the mouse over an empty tube. Mice were sacrificed by 5% isoflurane inhalation overdose using a chamber or cervical dislocation. To collect blood via heart right ventricular puncture, a 21-gauge needle coupled to a 1-mL syringe was used. Blood used for hematology was collected into EDTA-coated collection tubes (Greiner Bio-One #450531), and blood used for clinical chemistry measurements was collected into lithium-heparin tubes (Sarstedt #41.1503.015). For clinical chemistry analyses, plasma was isolated after centrifugation of the blood at 2000 ***g*** for 10 min at 4°C. Organs that were used for mRNA, ELISA, and iron measurements were harvested, washed in ice-cold PBS, flash-frozen in dry ice, and kept at −80°C until analyses. Kidneys used for histological analyses were fixed in 4% paraformaldehyde.

### Capillary hematocrit

A fraction of the EDTA blood collected was transferred to a hematocrit glass capillary (Hirschmann #9100275), and one of the extremities was sealed. The tubes were centrifuged at 13,000 rpm for 10 min using a hematocrit centrifuge (Hettich #2010). The red blood cells (RBCs) percentage was calculated from total volume measured with a digital caliper (Wabeco #11320).

### Hematology

EDTA tubes containing blood were kept at room temperature to preserve cell morphology until cell counting that took place in less than 4 hours after sampling. Blood cell analysis was carried out with an automated hematology analyzer (IDEXX #ProCyte DX) at the animal phenotyping facility of the Max Delbrück Center for Molecular Medicine, Berlin.

### Tissue non-heme iron level quantification

The liver, spleen, kidney, and duodenum were dried at 95°C overnight and weighed. One M nitric acid and ceramic beads were added to the samples. Tissues were homogenized using a FastPrep 24 homogenization device (MPI #116004500) and incubated at 65°C for~20 hours. Tubes were centrifuged at 10,000 ***g*** for 10 minutes at room temperature. The supernatant was transferred to a new tube and used for iron measurements. Iron levels in dry tissue extracts were measured with a colorimetric ferrozine-based assay (Roche #03183696122). Tissue iron levels were calculated by plotting the absorbance values against a FeCl_3_ standard curve.

### Plasma clinical chemistry

Plasma concentrations of iron, ferritin, transferrin, unsaturated iron-binding capacity (UIBC), urea, and creatinine (CRE) were measured using a clinical chemistry analyzer (Beckman Coulter #AU480). The total iron-binding capacity (TIBC) was calculated as (TIBC = iron + UIBC). The transferrin saturation percentage was calculated as (transferrin saturation = iron/TIBC * 100).

### Plasma and urine hepcidin quantification

Hepcidin was quantified using a commercially available ELISA kit (Elabscience #E-EL-M0671). Hepcidin levels are expressed as ng/mL in plasma, ng/mg protein, and ng/mg CRE in urine. Sample preparation, measurements, and calculations were performed following instructions provided by the company. Urine creatine concentration was quantified using a clinical chemistry analyzer (Beckman Coulter #AU480). Total proteins in liver extracts were quantified using a bicinchoninic acid assay kit (Sigma #BCA1-1KT).

### mRNA quantification by RT-qPCR

The mRNA levels of target genes were quantified in tissues after total RNA isolation and cDNA library preparation.

#### RNA extraction and cDNA synthesis

The liver, spleen, duodenum, and kidney were transferred to tubes containing ceramic beads and 1 mL of TRIzol (Invitrogen #15596018). Tissues were homogenized using a FastPrep 24 homogenization device (MPI #116004500). Possible contaminating genomic DNA was digested using DNase I (Sigma, #04716728001). For DNase I incubation and denaturation, 5–10 µg of RNA was used following the recommendations provided by the manufacturer. cDNA was prepared from the DNase I-treated RNA. Two µg of RNA was incubated with random hexamers and M-MLV reverse transcriptase (Promega #M170B) according to the instructions provided. All RNA measurements were done using a NanoDrop™ 1000 (Thermo Fischer # ND-1000).

### mRNA quantification

cDNA was diluted to a final concentration of 1 ng/µL, in nuclease-free water, for RT-qPCR application. All RT-qPCRs were done using SYBR green reagent mix (Promega #A6010) following manufacturer’s instructions. A QuantStudio™ 5 device (Thermo Fischer #A28140) was used for RT-qPCR cycling and gene expression analyses. The *C*t values obtained in the exponential phase of amplification from the gene of interest and the housekeeping genes *18 s, Actb*, *Polr2a* were used to calculate the relative gene expression to the control group. For calculations, the method of Livak and Schmittgen (2^−ΔΔCT^) was used. Sequences of specific primers targeting the mRNA of the genes of interest are displayed in, Supplementary Table S1.

### Kidney histological analyses

After fixation in 4% buffered paraformaldehyde, kidneys were dehydrated with ascending ethanol concentrations, embedded in paraffin, sectioned into 5-μm coronal sections using a rotary microtome (Thermo Fisher Scientific), mounted on SuperFrost Plus slides (Thermo Fisher Scientific), and dried overnight at room temperature until staining. Prior to staining, organ sections were deparaffinized and rehydrated in xylene and descending concentrations of ethanol. Kidney sections were stained with hematoxylin–eosin (Sigma-Aldrich), and images were acquired using a BZ-X800 microscope (Keyence).

### Data and statistical analyses

For all experiments, randomization was applied and blinding whenever possible. The number of biological replicates is represented in the bar graphs by scatter plot or indicated in the table legend. The minimum sample size was determined by power analyses. As parameters for power analysis, type I error was assumed at a significancy level of 0.05 and a power of 80%. The main parameter for power analysis was the hematocrit. No data replicates (mice) were excluded during the statistical processing. Statistical analyses were only performed in groups containing at least six independent replicates (mice) using Graph Pad Prism. Data are presented as mean  ±  SD, including scattered plots in bar graphs. Two-tailed paired Student’s *t* tests were used to test differences among two independent groups that were tested for normality. For all statistical tests used, a *P* < 0.05 was considered statistically significant.

## Results

### The anemic phenotype of Agt-KO

Initially, a capillary-based hematocrit was used to confirm anemia in Agt-KO. Male and female Agt-KO presented anemia as previously demonstrated in other studies using this and other RAS-deficient lines (Supplementary Figure S1A and S1B).This finding was confirmed in male mice with an automated cell counting device (Table 1). Agt-KO mice presented reduced RBC counts. Interestingly, the RBC size (mean corpuscular volume (MCV)) was also reduced in Agt-KO characterizing microcytic anemia. Furthermore, hemoglobin (HGB) and RBC hemoglobin content (mean corpuscular hemoglobin (MCH)) were found decreased in Agt-KO mice. However, the numbers of reticulocytes, platelets (PLTs), and white blood cells (WBCs) were unaltered in comparison with controls. All hematological parameters were measured in females, and the same parameters altered in male Agt-KO were also found altered in female Agt-KO (Supplementary Table S2).

**Table 1:** Hematology of Agt-KO.

Parameter, unit	Agt-Het, *n* = 7	Agt-KO, *n* = 7
Hematocrit, %RBC	50.0 ± 5.0	38.0 ± 4.0***
RBC, M/uL	10.3 ± 0.7	8.3 ± 0.8***
MCV, fL	48.3 ± 1.4	46.1 ± 1.1**
Hemoglobin, g/dL	14.9 ± 1.1	11.7 ± 1.2***
MCH, pg	14.5 ± 0.3	13.8 ± 0.3***
MCHC, g/dL	29.9 ± 0.7	29.8 ± 0.9
Reticulocytes, K/uL	503 ± 38.5	436 ± 88.8
Platelets, K/uL	1032 ± 149	1092 ± 114
WBC, K/uL	5.0 ± 1.0	4.5 ± 1.2

Values are mean ± SD **P<0.01, ***P<0.001 vs. Agt-Het (Student’s t test).

RBC, red blood cells. MCV, mean corpuscular volume. MCH, mean corpuscular hemoglobin. MCHC, mean corpuscular hemoglobin concentration. PLT, platelets. WBC, white blood cells.

### The iron balance in Agt-KO is disrupted

**Figure 1:**
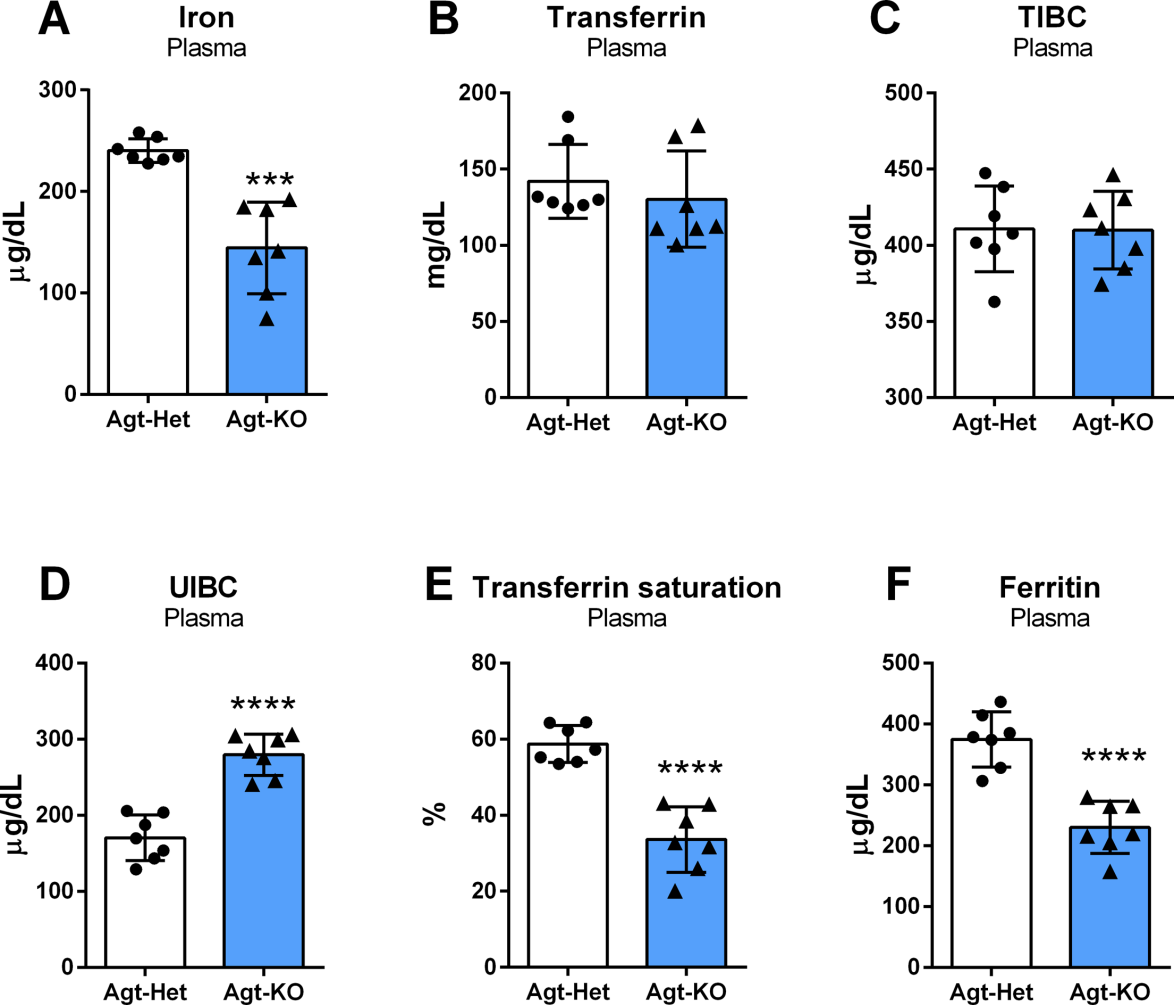
Plasma iron homeostasis. Quantification of plasma iron (**A**) and transferrin (**B**). Plasma calculated TIBC, total iron binding capacity (**C**). Plasma UIBC, unsaturated iron-binding capacity (**D**). Transferrin saturation in plasma (**E**). Ferritin levels in plasma (**F**). Values are mean ± SD ***P<0.001, ****P<0.0001 vs. Agt-Het (Student’s *t* test).

After finding a decreased MCV in the Agt-KO mice, we decided to quantify plasma iron and other parameters relevant for iron body homeostasis. Strikingly, plasma levels of iron were reduced in Agt-KO, which is in line with the microcytic anemia phenotype detected by the hematological analyses (Figure 1A). Plasma transferrin levels and the calculated TIBC were not different from controls (Figure 1B and C). However, the UIBC was found increased in Agt-KO (Figure 1D). Likewise, the calculated transferrin saturation was reduced in Agt-KO (Figure 1E). Finally, plasma ferritin levels were decreased in Agt-KO indicating tissue iron deficiency (Figure 1F).

**Figure 2:**
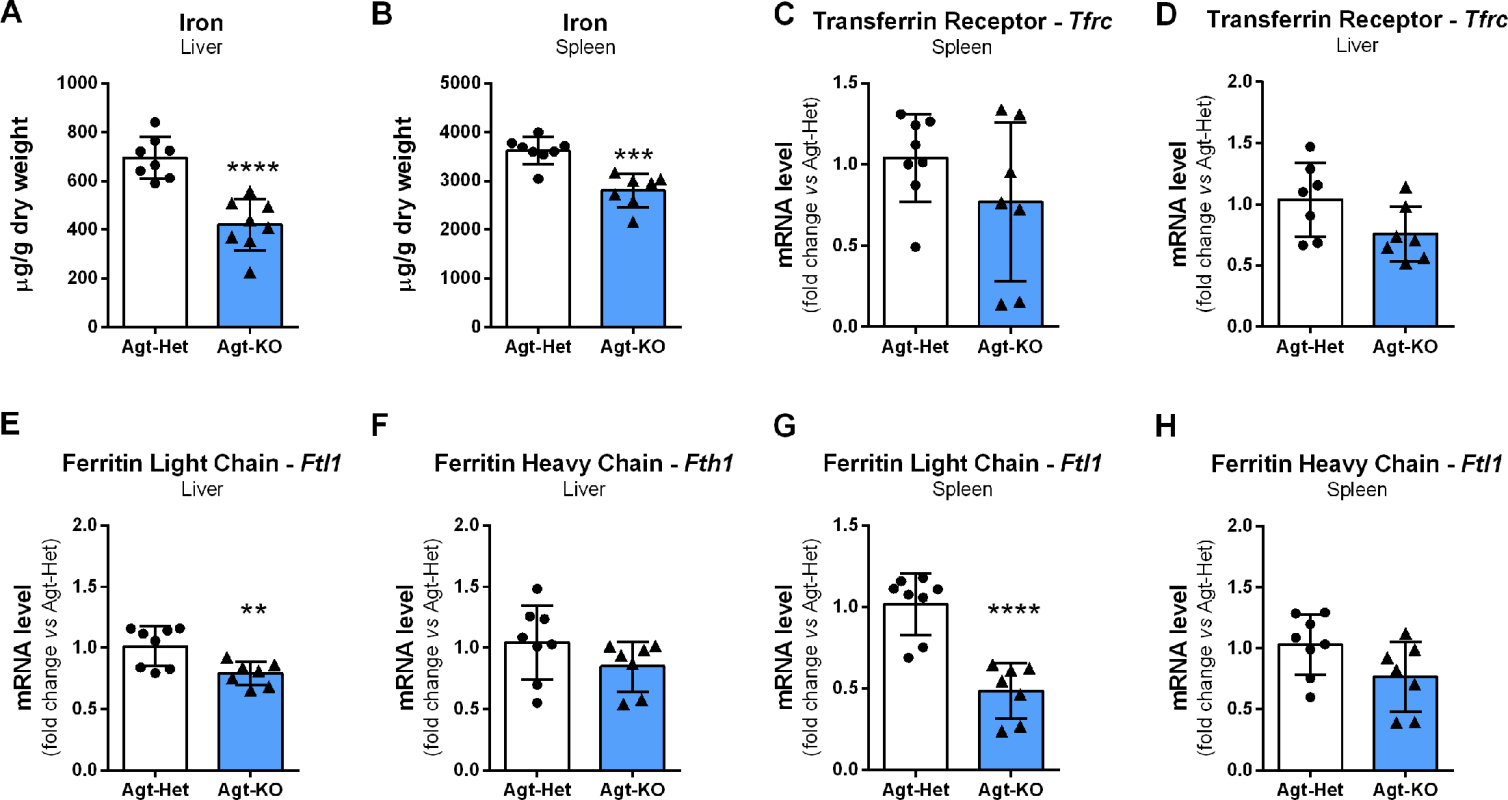
Tissue iron homeostasis. Quantification of iron content in liver (**A**) and spleen (**B**). Transferrin receptor (*Tfrc*) mRNA expression in liver (**C**) and spleen (**D**). Hepatic gene expression of light (*Ftl1,* **E**) and heavy (*Fth1,* **F**) chain ferritin. Splenic gene expression of light (*Ftl1,* **G**) and heavy (*Fth1,* **H**) chain ferritin. Values are mean ± SD **P<0.01, ***P<0.001, ****P<0. 0001 vs. Agt-Het (Student’s *t* test).

Liver and spleen are the most relevant tissues stocking iron for body iron homeostasis maintenance, and because the tissue iron marker ferritin was reduced in plasma of Agt-KO, the iron levels in these organs were quantified. The amount of iron in these tissues was reduced in Agt-KO corroborating the plasma ferritin findings (Figure 2A and B). In addition, the mRNA of the ferritin light chain encoding gene *Ftl1* was found down-regulated in liver and spleen of Agt-KO (Figure 2E and G). The mRNA expression of ferritin heavy chain, *Fth1* (Figure 2F and H), and the transferrin receptor, *Tfrc* (Figure 2C and D), was not altered in the spleen and liver of Agt-KO.

Plasma iron and other parameters measured in males were also quantified in the plasma of females. Of the parameters found altered in males, only ferritin was not different from controls, others were also altered in females (Supplementary Figure S2 A-F). Additionally, the levels of iron in the liver and spleen were quantified in females. As predicted by plasma ferritin quantification, the hepatic and splenic iron levels were not altered in females, (Supplementary Figure S2 G and H).

### Augmented plasma hepcidin causes iron deficiency in Agt-KO

**Figure 3:**
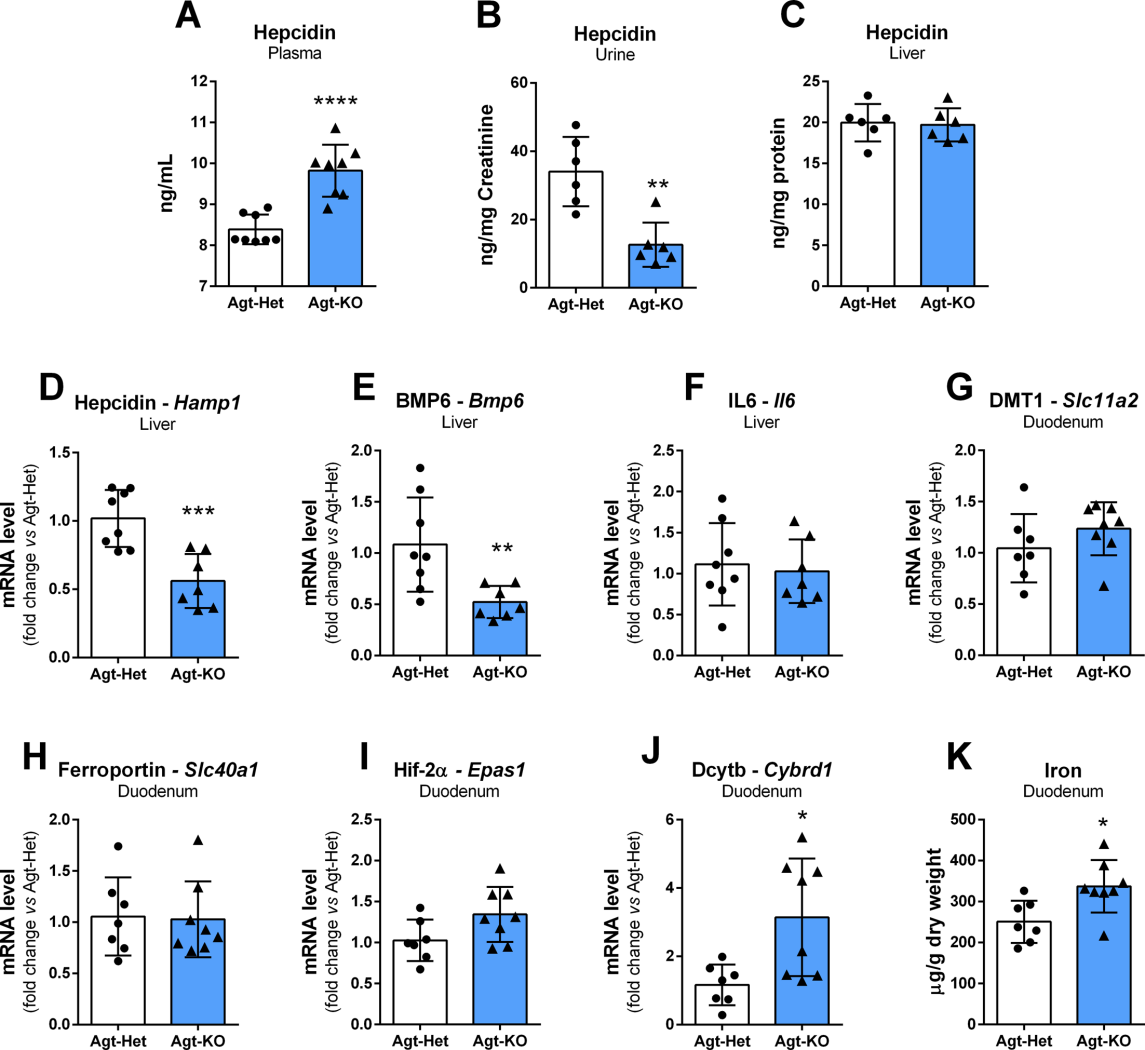
Expression of hepcidin and duodenal iron transporters. Plasma (**A**), urine (**B**) and liver (**C**) hepcidin quantification by ELISA. Hepatic hepcidin (*Hamp1*) (**D**), BMP6 (bone morphogenetic protein 6, *Bmp6*) (**E**), and IL6 (interleukin-6, *Il6*) (**F**) mRNA levels. Divalent metal transporter 1 (DMT1, *Slc11a2*) (**G**), ferroportin (*Slc40a1*) (**H**), hypoxia-inducible factor-2 alpha (Hif-2α, *Epas1*) (**I**) and duodenal cytochrome B (Dcytb, *Cybrd1*) (**J**) mRNA levels in duodenum. Quantification of iron in duodenum (**K**). Values are mean ± SD *P<0.05, **P<0.01, ***P<0.001, ****P<0. 0001 vs. Agt-Het (Student’s *t* test).

The plasma levels of hepcidin, the major hormone controlling iron absorption, were quantified by ELISA. Agt-KO presented increased hepcidin levels which explains the iron deficiency in this line (Figure 3A). Interestingly, Agt-KO urine hepcidin measurements showed reduced hepcidin contrasting the findings in plasma (Figure 3B).Circulating hepcidin is produced by hepatocytes, and therefore, we quantified hepcidin protein and mRNA in the liver by ELISA and RT-qPCR, respectively. Surprisingly, hepcidin protein levels were not changed in the liver of Agt-KO (Figure 3C), but the mRNA was down-regulated in the liver of Agt-KO (Figure 3D). In agreement with the liver hepcidin mRNA data, the mRNA levels of the major regulator of hepcidin expression that responds to plasma iron levels, BMP6 (bone morphogenetic protein 6), were also down-regulated (Figure 3E). Interleukin-6 is also known to modulate hepatic hepcidin expression in case of pathological inflammation, but the expression of this cytokine was not different from controls (Figure 3F). Altogether, Agt-KO responds to iron deficiency by decreasing liver hepcidin production via the canonical BMP6 pathway. However, the peptide hormone accumulates in the blood, causing iron deficiency despite its lower hepatic gene expression.

Next, we assessed duodenal iron uptake by measuring the mRNA levels of key proteins involved in this process by RT-qPCR. There were no alterations in *Slc11a2* encoding DMT1, divalent metal transporter 1 (Figure 3G), and *Slc40a1* encoding ferroportin (Figure 3H). Furthermore, the gene expression of the major transcription factor controlling ferroportin and DMT1 expression hypoxia-inducible factor-2 alpha (Hif-2α) was not altered in duodenum of Agt-KO mice (Figure 3I). We also quantified *Cybrd1* mRNA in duodenum that encodes duodenal cytochrome B (Dcytb), a key enzyme for iron uptake. This mRNA was increased in Agt-KO indicating a compensatory mechanism to overcome the iron deficiency (Figure 3J). Finally, duodenum iron levels were quantified and found to be increased in Agt-KO, suggesting a low export of iron into the circulation (Figure 3K).Ferritin light and heavy chain mRNAs were not altered in duodenum of Agt-KO (Supplementary Figure S3 A and B).

### Renal function of Agt-KO

**Figure 4:**
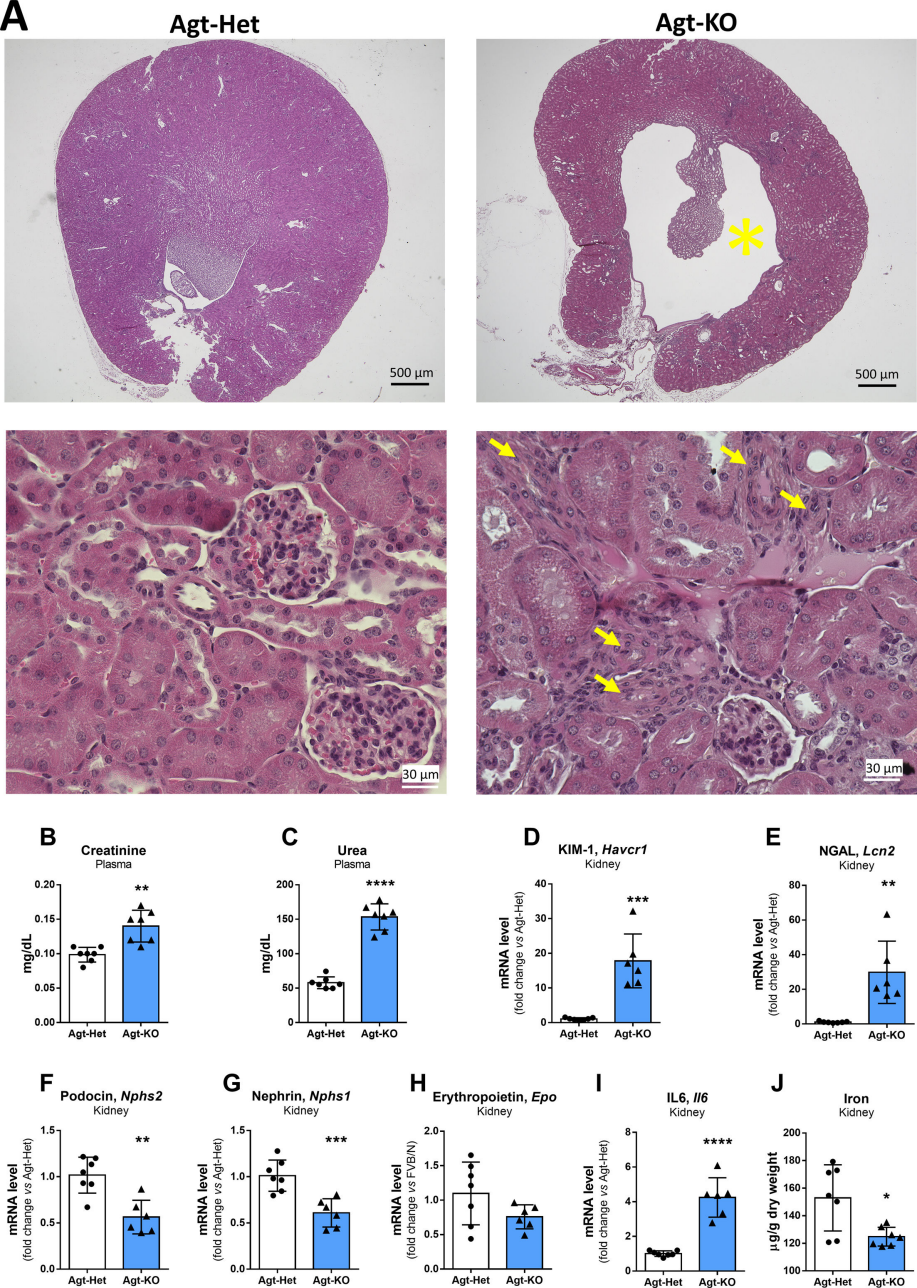
Renal function, damage and iron/erythropoietin homeostasis. Representative kidney coronal sections of male mice stained with hematoxylin and eosin. Agt-KO mice presented hydronephrosis (yellow asterisk) and marked thickening of interlobular arteries (yellow arrows) (**A**). Plasma quantification of creatinine (**B**) and urea (**C**). mRNA quantification of the renal damage markers KIM-1 (**D**), NGAL (**E**), Podocin (**F**), and Nephrin (**G**) in kidneys. Renal erythropoietin (*Epo*) (**H**) and IL-6 mRNA quantification (**I**). Quantification of iron content in kidney (J). Values are mean ± SD *P<0.05, **P<0.01, ***P<0.001, ****P<0. 0001 vs. Agt-Het (Student’s *t* test).

Agt-KO and other rodent lines lacking either Ang II production or AT1Rs, AT1aR, and AT1bR develop morphological and functional renal problems including reduced GFR. These findings were confirmed in these mice by histological analyses that revealed hydronephrosis and thickening of the renal arteries (Figure 4A), and measurements of the renal function markers (urea and CRE) in plasma. Both markers were elevated in male Agt-KO (Figure 4B and C). Females presented only elevated levels of urea (Supplementary Figure S4), and the increase was less pronounced than in males (Figure 4C and Supplementary Figure S4B). Overall, these parameters indicate that Agt-KO males develop a more severely impaired GFR function in comparison with age-matched females. Furthermore, the mRNA levels of kidney damage markers were quantified in the kidney of Agt-KO males. Corroborating severe kidney damage, KIM-1 and NGAL mRNA levels were found increased while Podocin and Nephrin mRNA levels were reduced in Agt-KO kidneys Figure 4E–G). Renal erythropoietin expression was not altered in Agt-KO (Figure 4F). Renal mRNA of IL-6 was found increased in Agt-KO samples (Figure 1I). Finally, kidney iron content was quantified in males, and it was reduced, like in the liver and spleen (Figure 1J).

## Discussion

**Figure 5:**
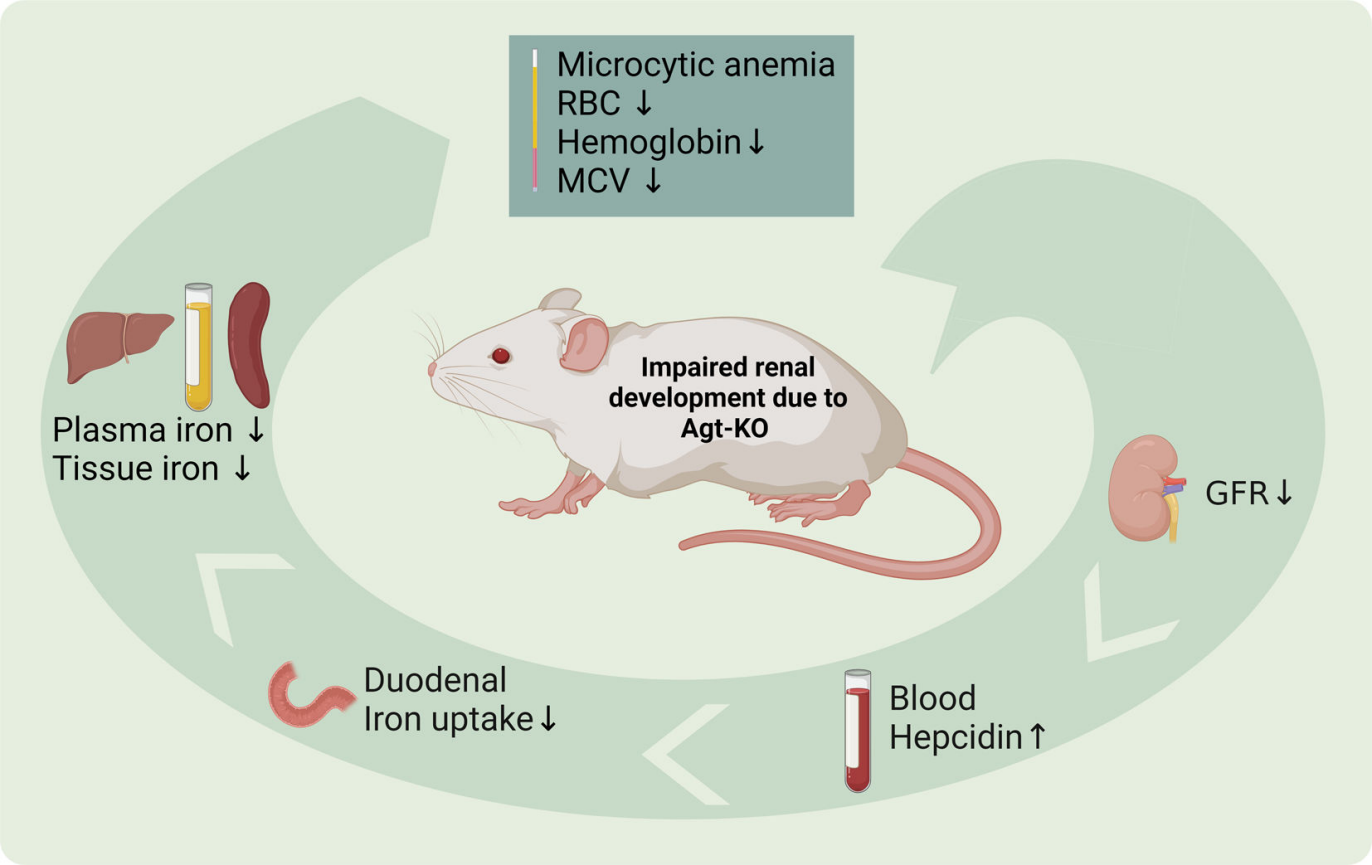
Scheme summarizing the findings of this study. The genetic deletion of angiotensinogen leads to impaired nephrogenesis followed by a reduction in renal glomerular filtration rate (GFR). Reduced GFR causes accumulation of hepcidin in the blood. High hepcidin dampens duodenum iron absorption that ultimately leads to systemic iron deficiency. Reduced iron supply contributes to the anemic phenotype of Agt-KO and likely other RAS-deficient models that present a severely impaired GFR.

Anemia is a well-recognized phenotype developed by long-term dampened RAS reactivity in experimental animals and humans [3,5,15,16]. In this study, we have further investigated this phenotype in mice with genetic deletion of the RAS precursor protein Agt (Agt-KO). The results of this study demonstrated that Agt-KO mice present a systemic iron deficiency, due to impaired renal development, leading to microcytic anemia in addition to the previously described RBC and hemoglobin reductions. Finally, the results indicate that the iron deficiency is driven by hepcidin accumulation in blood due to decreased GFR (Figure 5).There were several reports in which anemia has been demonstrated in RAS-deficient mice including ACE knockout [4], Agt-KO [5], renin knockout [5,12], and double AT1aR/AT1bR [5] receptor knockout. These studies collectively demonstrate that the Ang II/AT1R axis is relevant for erythropoiesis modulation *in vivo*. Moreover, deletional mutations in the *ACE* and *AGTR1* genes encoding the human ACE and AT1R, respectively, lead to anemia in humans demonstrating a conserved role [17,18]. Previous studies suggest that the modulatory mechanisms of Ang II on erythropoiesis occur by mechanisms operating by at least three levels. Ang II fosters the survival of bone marrow erythroid progenitors by direct binding to its AT1Rs, renal erythropoietin stimulation, and bone marrow sympathetic nerve activity stimulation that facilitates bone marrow erythroid progenitor expansion [3].

Anemia of RAS-deficient mice has been so far characterized by lower hematocrit, decreased RBC count, and decreased hemoglobin levels. Contrary to the present study, previous reports have demonstrated that the anemia of these animals is normocytic [4,5,12]. Therefore, the iron homeostasis was never an aspect deeply investigated in RAS-deficient mice. A single study quantified plasma iron levels in ACE-KO mice; this was the first report to demonstrate anemia in such animals. Their results indicated unaltered iron in serum, TIBC, and MCH in anemic ACE-KO mice [4]. One common feature of the aforementioned studies is that they all used *knockout* mice in the C57BL/6 genetic background strain. We have recently crossbred the originally mixed background strain Agt-KO to the FVB/N inbred background strain [14]. A great variability in the iron homeostasis among mice from different genetic background strains exists [19–21]. Comparing the FVB/N and C57BL/6 inbred strains, FVB/N presents tissue iron levels that are approximately five times higher than those of C57BL/6 [19]. Moreover, iron phenotype-related discrepancies were previously identified in models with identical genotypes on different background strains [21,22].

Previous studies could show that Ang II disturbs the iron homeostasis in various cell types in culture like human umbilical vein endothelial cells [23], human glomerular endothelium [24], rat neurons [25], bovine aortic endothelium [26], and *in vivo* in rat kidneys [27]. One particular study suggested that body iron homeostasis is disturbed in mice chronically treated with Ang II. Ang II-treated mice displayed lower levels of plasma iron and increased iron accumulation in macrophages accompanied by a lower hepatic hepcidin expression. Moreover, in this study, the authors found an increased mRNA expression of the duodenal iron transporters DMT1 and ferroportin due to increased duodenal HIF-2α [28]. In our study, however, the lack of Ang II in Agt-KO had no impact on the mRNA expression of HIF-2α, DMT1, and ferroportin in duodenum, therefore not explaining the iron deficiency.

The iron homeostasis phenotype of Agt-KO resembles the one developed by transgenic mice overexpressing hepcidin in a milder manifestation. Long-term increased hepcidin in the circulation as observed in Agt-KO and transgenic mice overexpressing hepcidin drives systemic iron deficiency [29,30]. Hepcidin is known to be the major hormone modulating iron uptake from the diet in the duodenum. Hepcidin binds and forces internalization-mediated degradation of the iron exporter ferroportin located at the duodenal basolateral membrane. The same ferroportin is expressed in tissues and macrophages playing a role to transfer iron stocked in cells into the circulation [31,32]. The life-long raised circulatory hepcidin likely depletes plasma and tissue iron levels, and the accumulated iron in the duodenum indicates a poor circulatory import of iron in Agt-KO as previously shown in other models with increased hepcidin [33,34].

Hepcidin is primarily eliminated from the body in the urine by the renal glomerular filtration. In fact, chronic kidney disease patients are susceptible to iron deficiency anemia development, especially if GFR is severely impaired as this group of patients accumulate high concentration of hepcidin in the circulation. Moreover, hemodialysis helps to remove the excess of hepcidin from the blood, and iron supplement increases the hemoglobin levels of such patients [35,36].

A decreased GFR has been previously demonstrated in RAS-deficient models including Agt-KO [14,37], renin, and ACE *knockout* lines [4,12,38,39]. The thickening of the renal arteries in Agt-KO, as observed in the present study, causes a reduction in the renal blood flow that ultimately reduces the GFR [40]. The GFR in Agt-KO mice has been previously demonstrated to be impaired by inulin clearance evaluation [37]. As surrogate markers, we quantified plasma CRE and urea that were elevated in Agt-KO mice confirming reduced GFR. Rodent models of CKD, including 5/6 nephrectomy, adenine overload, ischemia-reperfusion, and cisplatin treatment, also develop an impaired GFR and iron deficiency due to hepcidin accumulation in blood.

The adenine-induced CKD has been extensively used to study CKD-mediated iron deficiency. However, in this model, hepatic hepcidin mRNA is up-regulated by interleukin-6 as a consequence of direct and indirect CKD-mediated inflammatory mechanisms [31,41–45]. In Agt-KO IL-6, mRNA was increased in the kidney in line with our previous findings of increased immune cell infiltration in kidneys of Agt-KO [14]. However, the increased renal IL-6 does not influence hepatic hepcidin expression as in the adenine model but rather the low levels of plasma iron. In Agt-KO, low hepatic hepcidin expression is in line with the classical response to low circulatory iron that is sensed by the synovial endothelium and down-regulates the major factor controlling hepcidin expression, BMP6 [31,46]. The fact that plasma hepcidin levels are increased and urinary levels are reduced corroborates that its renal clearance is down-regulated and plasma half-live is likely up-regulated by the impaired GFR in Agt-KO mice. Curiously, the protein levels of hepcidin in the liver were not reduced as the mRNA, which may be explained by the increased hepcidin levels in the blood trapped in the liver which may compensate for decreased cellular levels of the protein. Altogether, Agt-KO experimentally confirms that hepcidin clearance relies on GFR as in previous preclinical models, hepatic hepcidin production was found increased not allowing to distinguish the impact of the reduced GFR. Thus, an impaired GFR as observed in CKD may raise plasma hepcidin levels even if hepatic hepcidin production is down-regulated.

This study demonstrates a previously uncharacterized phenotype in RAS-deficient mice, namely iron deficiency (Figure 5). The phenotype is a secondary consequence of a well-characterized improper renal development due to the lack of RAS activity during nephrogenesis. Interestingly, this model confirms that a poor GFR leads to hepcidin accumulation in blood confirming that patients with reduced GFR are prone to iron deficiency development.

**Clinical Perspectives**

- A functional renin–angiotensin system is known to be required during early nephrogenesis. Mice lacking angiotensin II have a reduced glomerular filtration rate and are anemic. The anemic phenotype was further characterized in such mice.
- In this study, we have identified that the reduced glomerular filtration rate of angiotensinogen-deficient mice leads to iron deficiency due to hepcidin accumulation in blood. Interestingly, mutant mice presented a down-regulation of the hepatic hepcidin mRNA, contrary to other preclinical chronic kidney disease models, demonstrating a major role of the kidneys in eliminating hepcidin.
- The findings are relevant for managing iron deficiency of chronic kidney disease patients that is also caused by increased levels of blood hepcidin.

## Supplementary material

Supplementary Figures and Tables

## Data Availability

The data that support the findings of this study are available from the corresponding author upon reasonable request.

## Abbreviations

ACE: angiotensin converting enzyme
AT1R: angiotensin type 1 receptor
Ang II: Angiotensin II
CKD: chronic kidney disease
CRE: creatinine
GFR: glomerular filtration rate
HGB: hemoglobin
MCH: mean corpuscular hemoglobin
MCV: mean corpuscular volume
PLT: platelets
RAS: renin-angiotensin system
RAS: renin-angiotensin system
RBCs: red blood cells
TIBC: total iron binding capacity
UIBC: unsaturated iron binding capacity
WBCs: white blood cells
