# Supplementary material for: Renal damage-induced hepcidin accumulation contributes to anemia in angiotensinogen-deficient mice
Source: Clin Sci (Lond). 2025 Feb 7;139(3):247–58. doi: 10.1042/CS20241789 (PMC12203995; doi:10.1042/CS20241789)
Supplement: Supplementary Figures and Tables [file CS-139-03-CS20241789-s001.docx]

Renal damage-induced hepcidin accumulation contributes to anemia in angiotensinogen-deficient mice

André F. Rodrigues^1,2,*^; Laura Boreggio^1,3^; Tetiana Lahuta^1,4^; Fatimunnisa Qadri^1^; Natalia Alenina^1,2^; Carlos C. Barros^1,5,6^; Mihail Todiras^1,7^ and Michael, Bader^1,2,3,8^

^1^Max-Delbrück-Center for Molecular Medicine in the Helmholtz Association (MDC), Berlin, Germany

^2^German Center for Cardiovascular Research (DZHK), Partner Site Berlin, Germany

^3^Experimental and Clinical Research Center, a cooperation between the Max-Delbrück-Center for Molecular Medicine in the Helmholtz Association and the Charité - Universitätsmedizin Berlin, Berlin, Germany

^4^Bogomoletz Institute of Physiology, Department of General and Molecular Pathophysiology, NAS of Ukraine, Kyiv, Ukraine.

^5^Nutrition Faculty, Federal University of Pelotas -UFPel, Pelotas, RS, Brazil

^6^Technological Development Center, Federal University of Pelotas -UFPel, Pelotas, RS, Brazil

^7^Nicolae Testemițanu State University of Medicine and Pharmacy, Chisinau, Moldova

^8^Institute for Biology, University of Lübeck, Lübeck, Germany

*Corresponding Author: Dr. André F. Rodrigues, Max Delbrück Center for Molecular Medicine, Robert-Rössle-Str. 10, 13125 Berlin, Germany. E-mail Address: [andrefelipe.rodrigues@mdc-berlin.de](mailto:anrodri@mdc-berlin.de)

**Table S1 – List of primer pairs used for mRNA quantification by RT-qPCR.**

| **Target, *Gene*** | | **Sequence (5' to 3')** | **Sense** | | **Amplicon (bp)** | |  |
| --- | --- | --- | --- | --- | --- | --- | --- |
| 18s, *Rn18s* | | TTGATTAAGTCCCTGCCCTTTGT | Forward | | 75 | |  |
|  | | CGATCCGAGGGCCTCACTA | Reverse | |  | |  |
| β-actin, *Actb* | | CTGGCCTCACTGTCCACCTT | Forward | | 61 | |  |
|  | | CGGACTCATCGTACTCCTGCTT | Reverse | |  | |  |
| BMP6, *Bmp6* | | AGAAGCGGGAGATGCAAAAGG | Forward | | 211 | |  |
|  | | GACAGGGCGTTGTAGAGATCC | Reverse | |  | |  |
| Dcytb, *Cybrd1* | | TTCCTGTCGGTGATCTTCGTG | Forward | | 203 | |  |
|  | | CCGGCATGGATGGATTTCATC | Reverse | |  | |  |
| DMT1, *Slc11a2* | | TACCTAGACCCAGGAAACATCG | Forward | | 132 | |  |
|  | | CACTCCAAGTCTCGCTGCAA | Reverse | |  | |  |
| Erythropoietin, *Epo* | | ACTCTCCTTGCTACTGATTCCT | Forward | | 123 | |  |
|  | | ATCGTGACATTTTCTGCCTCC | Reverse | |  | |  |
| Ferroportin, *Slc40a1* | | CTACCATTAGAAGGATTGACCAGCTA | Forward | | 82 | |  |
|  | | ACTGGAGAACCAAATGTCATAATCTG | Reverse | |  | |  |
| FTH, *Fth1* | | TGATGAAGCTGCAGAACCAG | Forward | | 105 | |  |
|  | | GTGCACACTCCATTGCATTC | Reverse | |  | |  |
| FTL, *Ftl1* | | AATGGGGTAAAACCCAGGAG | Forward | | 76 | |  |
|  | | AGATCCAAGAGGGCCTGATT | Reverse | |  | |  |
| Hepcidin, *Hamp1* | | AGGGCAGACATTGCGATACC | Forward | | 101 | |  |
|  | | GCAACAGATACCACACTGGGA | Reverse | |  | |  |
| HIF-2α, *Epas1* | | CTGAGGAAGGAGAAATCCCGT | Forward | | 161 | |  |
|  | | TGTGTCCGAAGGAAGCTGATG | Reverse | |  | |  |
| Interleukin-6, *Il6* | | CTGCAAGAGACTTCCATCCAGTT | Forward | | 70 | |  |
|  | | GAAGTAGGGAAGGCCGTGG | Reverse | |  | |  |
| KIM-1, *Havcr1* | | TCAGAAGAGCAGTCGGTACAAC | Forward | | 220 | |  |
|  | | TGTAGCTGTGGGCCTTGTAGT | Reverse | |  | |  |
| Nephrin, *Nphs1* | | GTGCCCTGAAGGACCCTACT | Forward | | 169 | |  |
|  | | CCTGTGGATCCCTTTGACAT | Reverse | |  | |  |
| NGAL, *Lcn2* | CCATCTATGAGCTACAAGAGAACAAT | | | Forward | | 89 | |
|  | TCTGATCCAGTAGCGACAGC | | | Reverse | |  | |
| Podocin, *Nphs2* | CTTGGCACATCGATCCCTCA | | | Forward | | 198 | |
|  | CGCACTTTGGCCTGTCTTTG | | | Reverse | |  | |
| RPB1, *Polr2a* | CAAGAGAGTGCAGTTCGGAGT | | | Forward | | 93 | |
|  | CCCTCCGTTGTTTCTGGGTATTT | | | Reverse | |  | |
| TFR, *Tfrc* | ATGCCGACAATAACATGAAGGC | | | Forward | | 136 | |
|  | ACACGCTTACAATAGCCCAGG | | | Reverse | |  | |

**Figure S1 – Capillary hematocrit.** Measured hematocrit in males (**A**) and females (**B**). Values are mean ± SD ****P*<0.001 *vs* Agt-Het (Student’s *t* test).

**Table S2 - Hematology of Agt-KO female.**

| **Parameter**, *unit* | **Agt-Het**, *n=7* | **Agt-KO**, *n=9* |
| --- | --- | --- |
| **Hematocrit**, *%RBC* | 47.1 ± 3.0 | 42.0 ± 1.7*** |
| **RBC**, *M/uL* | 9.5 ± 0.4 | 8.9 ± 0.3** |
| **MCV**, *fL* | 49.7 ± 1.3 | 47.0 ± 1.3*** |
| **Hemoglobin**, g*/dL* | 14.2 ± 0.6 | 12.7 ± 0.5**** |
| **MCH**, *pg* | 15.0 ± 0.2 | 14.3 ± 0.3*** |
| **MCHC**, *g/dL* | 30.2 ± 0.6 | 30.4 ± 0.5 |
| **Reticulocytes**, *K/uL* | 496 ± 97 | 409 ± 146 |
| **Platelets**, *K/uL* | 973 ± 106 | 1087 ± 181 |
| **WBC**, *K/uL* | 3.8 ± 0.8 | 4.37 ± 1.0 |

Values are mean ± SD ***P*<0.01, ****P*<0.001, ****P*<0.0001 vs Agt-Het (Student’s t test). RBC = red blood cell, MCV = mean corpuscular volume, MCH = mean corpuscular hemoglobin, MCHC = mean corpuscular hemoglobin concentration, PLT= platelets, WBC = white blood cell.

**Figure S2 – Plasma iron and iron homeostasis parameters, and tissue iron in females.** Iron levels in plasma (**A**). Transferrin levels in plasma (**B**). Plasma calculated TIBC, total iron binding capacity (**C**). Plasma UIBC, unsaturated iron binding capacity (**D**). Transferrin saturation in plasma (**E**). Ferritin levels in plasma (**F**). Concentration of iron in liver (**G**) and spleen (**H**). Values are mean ± SD **P*<0.05, ***P*<0.01 *vs* Agt-Het (Student’s *t* test).

**Figure S3 – Duodenal ferritin expression in males.** Light chain ferritin, Ftl1 (**A**) and heavy chain ferritin, Fth1 (**B**) mRNA quantification in duodenum. Values are mean ± SD.

**Figure S4 – Renal function markers in females.** Creatinine **(A)** and urea **(B).** Values are mean ± SD. *****P*<0.0001 *vs* Agt-Het (Student’s *t* test).
